# Hepatitis E Virus in Industrialized Countries: The Silent Threat

**DOI:** 10.1155/2016/9838041

**Published:** 2016-12-14

**Authors:** Pilar Clemente-Casares, Carlota Ramos-Romero, Eugenio Ramirez-Gonzalez, Antonio Mas

**Affiliations:** ^1^Centro Regional de Investigaciones Biomédicas, Universidad de Castilla-La Mancha, 02008 Albacete, Spain; ^2^School of Pharmacy, Universidad de Castilla-La Mancha, 02071 Albacete, Spain; ^3^School of Medicine, Universidad de Castilla-La Mancha, 02008 Albacete, Spain

## Abstract

Hepatitis E virus (HEV) is the main cause of acute viral hepatitis worldwide. Its presence in developing countries has been documented for decades. Developed countries were supposed to be virus-free and initially only imported cases were detected in those areas. However, sporadic and autochthonous cases of HEV infection have been identified and studies reveal that the virus is worldwide spread. Chronic hepatitis and multiple extrahepatic manifestations have also been associated with HEV. We review the data from European countries, where human, animal, and environmental data have been collected since the 90s. In Europe, autochthonous HEV strains were first detected in the late 90s and early 2000s. Since then, serological data have shown that the virus infects quite frequently the European population and that some species, such as pigs, wild boars, and deer, are reservoirs. HEV strains can be isolated from environmental samples and reach the food chain, as shown by the detection of the virus in mussels and in contaminated pork products as sausages or meat. All these data highlight the need of studies directed to control the sources of HEV to protect immunocompromised individuals that seem the weakest link of the HEV epidemiology in industrialized regions.

## 1. Introduction

Hepatitis E virus (HEV) is the causative agent of hepatitis E, the most frequent enterically transmitted hepatitis in the world and currently considered an important public health problem worldwide [1, 2]. It is also the most common acute viral hepatitis, causing about 50% of acute hepatitis in developing countries [2–4]. According to WHO, one-third of the world population has been exposed to HEV [5].

The virus was discovered after an outbreak of hepatitis of unknown etiology in Kashmir valley (India), in 1978 [6], and it was molecularly characterized in 1983 [7]. HEV belongs to the family Hepeviridae that includes 2 genera:* Orthohepevirus* (with species that infect mammals and birds) and* Piscihepevirus* (infecting trout) [8]. Four genotypes have been isolated from humans and they can be classified according to their epidemiological characteristics and survival strategies. Genotypes 1 and 2 are responsible for human infections exclusively, while genotypes 3 and 4 can infect humans and other animals [5–9].

Epidemiology of HEV infection is more complex than initially thought because it includes two distinct epidemiological patterns of disease, with different characteristics [10]. Genotype 1 strains have been identified in Asia and Africa but also circulate in Cuba and Venezuela. These are frequently responsible for cases of acute hepatitis E imported to Europe by international travelers, mainly from Asia. Genotype 2 strains are found in Africa and in Mexico. Both genotypes are transmitted through fecally contaminated water, infect humans, and are associated with outbreaks [11]. Genotype 3 strains are found worldwide and affect wild and domestic mammals. This genotype causes sporadic infections in humans through zoonotic transmission or consumption of contaminated food [9]. It is responsible for most of the autochthonous cases in Europe [12]. Strains within genotype 4 are very similar to those of genotype 3, also constituting a zoonosis. Although some autochthonous cases caused by this genotype have been reported in Europe, the frequency is much higher in Southeastern Asia and the Far East [11].

The main routes of transmission of HEV are consumption of contaminated water and food and vertical and person-to-person transmission. Parenteral transmission is also possible [4, 13].

The diseases caused by the different genotypes of HEV share clinical features with other acute viral hepatitis [14]. A wide range of clinical manifestations, from asymptomatic or subclinical to acute liver failure, can be observed [15]. The current rate between infection and disease is still unknown, but it is accepted that asymptomatic infection is the most common scenario [16]. The data from an outbreak on a cruise ship caused by genotype 3 showed that 67% of the infected people were asymptomatic [17]. Patients with symptomatic disease usually suffer from jaundice, anorexia, abdominal pain, and hepatomegaly. Fever, nausea, and vomiting occur less frequently [14].

Mortality caused by HEV (0.2–4% in epidemics) is due to acute or subacute liver failure [4]. Among pregnant women higher incidence and severity, including fulminant hepatic failure, have been reported associated with genotype 1 strains. Mortality in this group is increased, especially in the third trimester, reaching 25–30% in areas such as northern and central India and Pakistan [2–5]. Genotype 1 has also been associated with abortion, low birth weight, and increased perinatal mortality [13], but there is scarce data about the potential of other genotypes to cause these complications [18].

Although it is mostly an acute infection, cases of chronic infection, defined as the persistence of RNA in serum or stool for 6 or more months, have been reported among immunocompromised patients [5, 19]. These patients are at higher risk of fulminant hepatitis failure, chronification of the infection, and rapid evolution to cirrhosis [20–22].

Several extrahepatic manifestations associated with HEV infection by genotypes 1 and 3, such as neurological disorders, have been described [23]. According to Kamar et al. [24] 5.5% of the studied patients with acute and chronic HEV genotype 3 infections developed neurological injury. These neurological disorders include Guillain-Barré syndrome, neuralgic amyotrophy, and encephalitis/meningoencephalitis/myositis [23]. The pathophysiology of these injuries may be due to the immune response triggered by the virus or to the direct neurotrophy of the HEV. Kidney injury and hematological manifestations have also been described in HEV-infected patients [23].

## 2. Seroprevalence of HEV in General Population and Blood Donors in Europe

Although it was initially considered an enteric infection confined to developing countries, HEV has been frequently detected in industrialized countries beyond those cases imported by travelers from endemic areas since the early 2000s. In fact, it is currently considered an emerging pathogen in developed areas [59].

In European countries, as in other developed areas, values of seroprevalence against HEV are difficult to interpret, probably due to the lack of genotype-specific assays and the wide diversity of methods employed to detect antibodies against the virus, which vary in sensitivity and specificity [4, 36, 60–62]. In fact, the analysis of a given population by using different assays usually reports very variable results [32, 36, 37, 39, 60, 62]. Sauleda et al. [36], after testing more than 1000 serum samples in Catalonia (Spain) with two different commercial ELISA assays, obtained discordant values (19.96% and 10.72%). Similar results have been reported by other authors [32, 37, 39]. Probably only data obtained with the same assay should be compared [32, 37]. In general, the Wantai assay gives higher seroprevalence values compared to others [62].

HEV seroprevalence varies among European countries (Table 1). Some values reported for general population are 13% in UK [25], 2.7% in Italy [51], 1.9% in Netherlands [43], 9.3% in Sweden [45], 16.8% in Germany [38], 1.5% in San Marino [63], and 7.3% in Spain [34]. Variations are also found within countries. In France, regional differences have been observed, with values ranging from 8.0 to 86.4% [30]. In some cases, for example, Spain, seroprevalence has been reported by different authors varying from 0.8 to 7.3% [34, 35, 64], though it is unclear whether those differences may be attributed to the assays used or to geographical differences.

As expected, values are also variable for blood donors and can be as divergent as 4.7% in Scotland and 52.2% in southwestern France. Some seroprevalence values reported for this group in different European countries are summarized in Table 1. The most interesting scenario is again southern France, where the overall seroprevalence among blood donors is 39.1% but ranges from 21.9% to 71.3% depending on the geographical area [65]. Regional differences among blood donors have also been reported in other areas in Europe, such as the Netherlands [33].

In France, Mansuy et al. [30] recently reported increased seroprevalence values when comparing blood donor samples from early 2000s and 2011-12. A similar trend was observed in the Netherlands [66]. On the contrary, some authors have reported a strong birth cohort effect in HEV seroprevalence in Denmark, with declining seroprevalence values over the last 30 years [32], probably due to lower exposure rates as a consequence of the improvement in sanitation and general living conditions. Very similar results have also been published in Germany [37].

The prevalence of anti-HEV antibodies raises with age [25, 30, 33, 38, 67]. Slot et al. [33] estimated a 1.05% increase of seroprevalence per year after the age of 30 among blood donors in the Netherlands. In Austria, values ranged from 8.1% in the group aged 18-19 years to 57.5% in the group aged 50–60 years [68]. The observed increasing prevalence associated with age can be due either to a uniform exposure throughout life or to a greater exposure of the older groups [36]. IgG prevalence among children is generally less than 5% [25, 31, 35, 69–71] and infection in this group is often asymptomatic [36].

A higher seroprevalence among male individuals has also been widely reported. Mansuy et al. [30], after analyzing more than 10000 blood donor samples, observed a significantly higher rate of exposure among men compared to women (25% versus 19.5%). Similar trends have been reported by several other groups [33, 34, 36, 70, 72]. On the contrary, other studies did not find significant differences by gender [40, 73, 74].

## 3. Acute and Chronic Hepatitis E

In Europe, 5–15% of the acute hepatitis of unknown origin are caused by HEV [67, 75–79]. In the 90s, the diagnosed patient would have probably traveled to an endemic area and come back to Europe with an imported HEV strain. In the late 90s and early 2000s, the first autochthonous cases of HEV infections by genotype 3 were reported [80–83]. Since then, similar cases locally acquired have been reported regularly throughout Europe [26, 67, 84–93]. Nowadays, the profile of the patient suffering from an acute E hepatitis is a man older than 40 with no travel history to endemic areas.

As expected, most of the acute hepatitis E cases reported in Europe are asymptomatic and self-limited infections. It has been reported that some symptomatic HEV infections may be misdiagnosed as drug-induced liver injury [76]. Although autochthonous acute hepatitis E cases in Europe are preferentially caused by genotype 3 strains, some genotype 4 autochthonous cases have recently been detected in France, Denmark, Italy, and Germany [90, 94–98]. Likewise, imported infections are also diagnosed, usually from immigrants or travelers coming back from Asia, Africa, and South America [67, 90, 91, 93, 99]. These infections are typically due to genotype 1 strains, although some cases caused by genotype 4 have also been identified [98, 100, 101]. In general, individuals with imported HEV infections are younger than those with locally acquired ones [84, 90].

Some cases of fulminant hepatitis have also been described [102–110]. Immunocompromised patients in Europe include HIV-infected patients [111–115], solid-organ transplant (SOT) recipients [15, 19, 116–126], and individuals under immunosuppressive therapy or with hematological diseases [126–131]. Chronic hepatitis E is caused by genotype 3 strains [9], although one recently described case has been associated with genotype 4 [132]. According to some authors, the reduction of immunosuppressive therapy (if possible) or treatments with ribavirin or pegylated-interferon plus ribavirin may induce the clearance of the virus [15, 114, 125, 131]. In Spain, one chronic HEV infection was identified in an immunocompetent man as reported by Gonzalez Tallon et al. [133].

Cases of neurological disorders associated with HEV infections have also been detected in Europe. van den Berg et al. [134] concluded that 5% of patients with Guillain-Barré syndrome in the Netherlands had an associated HEV infection. Acute HEV infection was also detected in 10% of patients with neuralgic amyotrophy in UK and the Netherlands [135]. Although Guillain-Barré syndrome and neuralgic amyotrophy are the most frequently reported neurological disorders, encephalitis, meningitis, peripheral neuropathy, or polyradiculoneuropathy has been also described in HEV-infected patients in Europe [24, 136–139].

## 4. HEV in Animals

HEV genotypes 3 and 4 have been identified in multiple species. In 1997, Meng et al. [140] reported for the first time the detection of the virus in pigs in the United States. Since then, HEV has been isolated from different species such as deer, wild boar, monkey, mongoose, rabbit, ferret, trout, bat, rat, chicken, kestrel, falcon, camel, and trout [141]. However, only pigs, wild boars, and deer are considered true reservoirs of the virus for humans [142]. Pigs, usually with subclinical infection, are considered the most important among them [9].

In Europe, high HEV seroprevalence has been detected in swine from Spain [143, 144], Italy [145–147], Norway [29], Denmark [148], UK [149, 150], Estonia [151], Germany [152, 153], or Switzerland [154, 155]. According to the seroprevalence data, 76% to 98% of Spanish swine farms have evidence of HEV presence [143, 144, 156, 157]. Some farms are virus-free, while in others up to 100% of the animals have detectable IgG levels [143, 144]. In Germany, Baechlein [158] showed that in 78.2% of the investigated farms at least one seropositive animal was identified, reaching 100% in some areas.

Seminati et al. [144] studied the dynamics of infection in a seropositive farm. They analyzed the presence of antibodies and viral RNA in different groups of age and concluded that circulation of HEV usually occurs at the end of the nursery or the beginning of the fattening period. It is widely accepted that breeding sows can play a role as HEV reservoirs and can transmit the virus to sucking piglets [144, 148, 156, 159, 160]. Pigs of less than 2 months are protected against HEV infection due to the presence of maternal antibodies [140, 161, 162]. HEV infection in pigs is subclinical, with only microscopic liver lesions [140, 163].

Although infection in pigs seems to occur at early stages in animal life, virus can be detected at all ages [156, 160, 162, 164]. On the other hand, RNA has been found in feces, liver, bile, and cecal content of animals at slaughter age, representing a risk of entrance of HEV into the food chain [149, 150, 157, 160]. Casas et al. [160] identified the virus in liver or bile of 3 animals at slaughter age 3 months after the first reports of their infection. If those cases were due to persistent infections in those organs or reinfections remains unknown, although the sequences in one of the animals were different enough to assume reinfection.

Wild boar and deer populations are also considered HEV reservoirs in Europe. In Spain the seroprevalence values among wild boars range from 26.3% to 57.4% [165–167]. Similar results were observed in Italy, (29.2% [147] and 40.7% [168]), Germany (41% [169]), Belgium (34% [170]), or Poland (44.4% [171]). Lower values have been observed among deer populations, for example, red deer (10,4% [172], 12,85% [165], 2–3.3% [173], 8% [174], and 1% [170]), roe deer (5.4–6.8% [173], 3% [170]), and moose [175].

HEV strains isolated from pigs, wild boars, and deer in Europe belong to genotype 3 [146, 149, 156, 165, 166, 168, 172, 176–178], although genotype 4 strains have also been detected in pigs in Belgium and Italy [95, 179, 180]. Swine HEV isolates are usually highly related to HEV sequences isolated from humans in the same area, that is, exhibiting even more similarities with human sequences than with swine sequences isolated from different regions [156, 157, 181]. Likewise similarities between HEV sequences isolated from wild boar, deer, and domestic pigs can be as high as 99%, which may be considered as further evidence of transmission between them [166, 167, 172].

Other animal species infected by HEV in Europe are rabbit, goat, rat, ferret, and mink, although phylogenetic studies mostly show high divergence with human HEV strains. Di Bartolo et al. [182] studied the presence of antibodies against HEV in farmed and pet rabbits in Italy, reporting seroprevalence of 3.4% and 6.56%, respectively, but no HEV RNA was found. In France, Izopet et al. [183] detected the virus in bile samples from farmed rabbits (7%) and in liver samples from wild rabbits (23%), with all the strains detected clustered and forming a clade clearly separated from genotypes 1 to 4. Furthermore, some of those rabbit strains were very similar to HEV strain from a man also from France [183]. HEV infecting rabbits has also been detected in Germany [184] and the Netherlands [185] and from a pet house rabbit in Italy [186].

Very recently, Di Martino et al. [187] described genotype 3 strains infecting goats in Italy. Antibodies against HEV had already been detected in goats (0.6%) in Spain, as well as in sheep (1.92%) and cats (11.11%) [188]. McElroy et al. [189] showed that dogs in the UK are also susceptible to HEV infection (seroprevalence of 0.8%).

Rat HEV strains have been detected in Germany, where a survey in different areas showed a broad geographical distribution of the virus in Norway rats with an overall seroprevalence of 24.5% [190] and in Denmark [191]. Stalin Raj et al. [192] detected HEV RNA in 9.3% of fecal samples collected from household pet ferrets without overt clinical signs. The sequences clustered with rat HEV.

Finally, the presence of HEV among birds has also been reported in Europe. Marek et al. [193] demonstrated a wide spreading of the avian HEV in chicken flocks in Europe. Peralta et al. [194] reported that 89.7% of the Spanish chicken flocks analyzed in their study had at least 1 seropositive animal. The isolated strains were similar to other avian HEV sequences previously isolated in USA and Canada, although forming a different genetic cluster. Reuter et al. [195] found HEV in common kestrels and red-footed falcons in Hungary. The sequence detected in a kestrel was more related to ferret and rats HEV isolates than to any other avian.

## 5. HEV in Environment and Food

Many viruses reach the environment and can infect people via water and food. HEV is excreted in feces [196] and has also been detected in urine [197, 198]. These viral particles can reach the environment and contaminate water sources and irrigated food. In fact, drinking contaminated water is the main mode of transmission of the virus in developing countries [142].

HEV can be detected in sewage. Multiple studies performed in Spain detected the presence of HEV in urban and slaughterhouse sewage [83, 181, 199–201]. Some of those studies isolated the virus from more than 30% of the raw urban sewage samples tested [181, 201]. The virus has also been detected in sewage from Italy [202, 203], France [204], or Norway [205]. Although sewage is treated before being released to the environment, conventional wastewater treatment does not completely remove all viruses [206]. Rusiñol et al. [206] studied the elimination of HEV during wastewater treatment by testing influent raw sewage and secondary and tertiary treated effluents from a wastewater treatment plant. Although the virus is present in raw sewage and secondary effluent, it was not detected in the tertiary effluent samples. Myrmel et al. [205] also detected HEV in secondary treated water.

HEV can be concentrated in some waste products generated during sewage and drinking water treatments (i.e., sludge and biosolid) [200, 207, 208]. Land application of this waste can contaminate water from aquifers and vegetables irrigated. Kokkinos et al. [209] reported the presence of HEV in 5% of the irrigation water samples tested from the leafy green vegetables production chain. In another study, the authors also detected 4 samples (3.2%) of fresh lettuce contaminated with the virus [210]. HEV has also been detected from frozen raspberries (1/38) [211]. Renou et al. [212] identified consumption of water from springs or private wells and vegetables from gardens irrigated with those water sources as risk factors for acquiring hepatitis E infection in France. In Slovenia, Steyer et al. [213] found HEV in surface water samples, one of them collected near a pig farm. In fact, the identity with other Slovenian swine HEV isolates in the area was 100%. The virus was also detected in a river sample in Italy [214] and the Netherlands [215].

Run-offs from animal facilities have been implicated in human HEV infections as the virus has been detected in manure and wastewater with animal fecal contamination [215, 216]. It is also present in slurry samples from pig manure composting plants in Spain and other European regions [164, 217, 218]. In the Spanish study [217], the authors concluded that composting process was effective in eliminating HEV from the slurries.

Routes of food contamination are diverse and include those already mentioned (i.e., application of organic wastes to agricultural land as fertilizer and contamination of water used for irrigation with fecal material) and also direct contamination by livestock, wild animals, and birds as well as postharvest issues such as worker hygiene [219]. The consumption of meat from HEV-infected animals is a source of HEV transmission to human and a public health concern [220]. In fact, some cases of direct transmission have already been documented in Europe [221, 222] and others have been indirectly associated with contaminated food [204, 223, 224]. Although the virus replicates in liver, it has also been detected in other tissues such as stomach, kidney, or multiple muscle masses [160, 225]. HEV have been detected in the food chain of pork products in the Netherlands [226], France [223, 227], Germany [228], Italy [176], Czech Republic [176], and Spain [176, 229]. This may not be uncommon if animals at slaughter age can be actively infected by the virus [149, 160]. Di Bartolo et al. [176] detected HEV, not only in packaged sausages and liver samples from processing sites and supermarkets in Spain, Italy, and Czech Republic but also from environmental samples collected in production farms and processing plants and at the points of sale in items such as knives, floor, belt surfaces, workers' hands, and toilets. HEV-positive samples were found more frequently at slaughterhouses than at processing and points of sale. In Canada, Nantel-Fortier et al. [230] recently found that movements of trucks and utility vehicles might play an important role in HEV dissemination on a slaughterhouse site and throughout the entire network.

The analysis of meat samples confiscated to passengers in the International Airport of Bilbao (Spain) on flights from non-European countries in 2012 and 2013 revealed that 53.3% of these samples were positive for HEV, including pork, poultry, beef, sheep, antelope, or kangaroo samples [229]. Contamination of food products during the manipulation via human handlers in the place of origin may explain the high percentage of HEV-positive samples among nonpork meat products found by the authors, some isolates being genotypes 1 and 2. The estimated viral load was very low and no data about infectivity was provided.

Coastal waters may also be contaminated by HEV leading to accumulation of the virus in the digestive tissues of shellfish due to their filter-feeding nature [220, 231] and processing interventions such as depuration do not completely remove viral particles [232]. Some studies have detected HEV in mussels or oysters. In Spain, Mesquita et al. [233] found that 15% of mussels batches tested from Galicia (Spain) were contaminated by the virus. In a similar study, Diez-Valcarce et al. [231] analyzed commercial mussels obtained at local retail stores in Spain and Finland, obtaining 6% of positive samples in the Spanish specimens. Analogously, HEV accumulation in shellfish has also been described in Scotland [234]. Thus contaminated bivalve mollusks suppose a health risk for people who eat them raw or slightly cooked [234, 235]. HEV outbreak on a cruise ship was associated with consumption of shellfish, although the source was not identified [17].

## 6. Routes of Transmission and Risk Groups

Travelers returning from HEV endemic areas were the first risk group considered in industrialized countries. In fact, although autochthonous strains are gaining increased importance, the imported cases are still frequently diagnosed, usually associated with genotype 1 strains [67, 88, 104, 108, 109, 236].

Consumption of contaminated water is the major route of transmission of the infection in developing countries, but this is not the case in industrialized countries, where sanitation conditions are supposed to be better. However, also in these areas, consumption of untreated water is also considered a risk for HEV infection [237]. In this study conducted in Spain, consumption of untreated water from water fountains near swine farms was associated with higher seroprevalence. Similarly, Mansuy et al. [30] found that drinking bottled water was associated with a lower risk of HEV IgG in France.

Exposition to HEV susceptible animals, mostly swine and wild game species, that is, wild boar and deer, is usually considered a risk factor for acquiring an HEV infection (Table 2). In Spain, Galiana et al. [237] compared the presence of IgG against HEV in people exposed to swine (farmers, handlers, and veterinarians) versus nonexposed individuals (controls) and found significantly higher values among the former (18.8% versus 4%), which confirmed the higher risk of acquiring HEV among people in contact with pigs. Similar results were obtained in the Netherlands and France [41, 43]. In Norway, increased seroprevalence has recently been reported in swine farm workers, but not among veterinarians [29]. In Spain, a case of acute hepatitis E in a slaughterhouse worker, probably infected after manipulating HEV-infected animals, was reported [238]. However, Meader et al. [239] did not identify farm contact with pigs as a risk factor for HEV infection.

Other professional and recreational activities have been associated with an increased risk of acquiring HEV such as hunters and forestry workers, suggesting that animal stools may provide an additional source of HEV infection among people with close contact with the forest environment [42, 49, 240]. In Turkey, agricultural workers who used untreated wastewater for irrigation showed a seroprevalence value of 34.8% compared to the 4.4% found in the control group [50]. On the contrary, in Germany, some studies concluded that sewage is not a source of infection for sewage workers [241, 242].

Consumption of raw or undercooked meat has also been suggested as a risk factor for HEV infection because of the following: (a) the virus is frequently isolated from animals at slaughter age and meat products at point-of-sale [149, 150, 176, 223, 227], (b) it is unknown how long the virus can survive in an infectious state in food products [243], (c) some studies indicate that HEV can survive and keep infectivity at temperatures used in some cooking regimens [244, 245], and (d) the consumption of raw or undercooked meat has been frequently identified as risk factor for HEV infection [30, 41, 65, 246]. In Spain, Riveiro-Barciela et al. [222] described the zoonotic origin of an acute hepatitis E in an immunocompromised patient after pork meat ingestion. Identical HEV sequences were isolated from the patient and a leftover frozen piece of meat. This transmission route provides a plausible explanation for the high seroprevalence observed in the general population without other risk factors and it is supported by the high similarity observed between human and animal HEV isolates from the same area [9, 140, 181, 247]. The transmission of the HEV by consumption of raw meat has also been demonstrated for meat products derived from wild boar [248] and deer [249] in Japan. Recreational hunting of wild boars and further consumption of their meats provide an ideal condition for the transmission of pathogens such as HEV [250, 251]. Consumption of shellfish is another risk factor described frequently in Europe [65, 231, 233].

In Spain, IgG seroprevalence among pregnant women varies from 0.6 to 5.4%, similar to the prevalence in general population [252–254]. IgM seroprevalence reported was 0.67% [254], suggesting that although subclinical infections exist, prevalence is very low. In France, authors conclude that HEV infection during pregnancy is rare even in areas with high seroprevalence [255]. In fact, only few cases of HEV infection during pregnancy have been reported in Europe [256, 257] and no complications were described. The predominance of genotype 3, which seems to be less virulent than other genotypes, is one of the possible explanations for the different outcome of the infection related to endemic areas [254]. One case of fulminant hepatitis E in a woman with oral contraceptive treatment was described by Mateos Lindemann et al. [103].

Immunocompromised subpopulations are also considered groups of risk for HEV. In Europe, reported seroprevalence among HIV ranges from 1% to 26% [52, 258–265] with some countries, for example, Spain, with values ranging from 9 to 26% depending on the study [52, 53, 259, 260, 266–268]. According to some authors, HIV infection seems to be a risk factor, not for acquiring an HEV infection, but for developing a chronic infection. Studies in Germany [269], Spain [53], and UK [270] reported no significant differences in HEV seroprevalence among HIV patients when compared to the general population. Pischke et al. [269] also concluded that the risk of developing chronic infection was very low if those patients were properly treated. Something similar was reported by other authors [270, 271]. In some studies, low CD4^+^ T cell counts have been associated with higher risk of HEV infection [260, 265]. Jardi et al. [259] concluded that the only factor associated with HEV IgG detection among HIV patients was the presence of liver cirrhosis (23% in patients with cirrhosis versus 6% in patients without cirrhosis), suggesting that individuals with cirrhosis are at a high risk of acquiring HEV infection [259]. This has also been reported by other authors [19, 260].

Solid-organ transplant (SOT) recipients are usually immunocompromised and, in addition to the fecal-oral route of transmission of the HEV, they can also get infected by administration of blood products and via the transplanted organ [272]. Some studies in Europe have reported cases of HEV infection after transplant, most of them asymptomatic. In France, Kamar et al. [19] reported that 14 out of 217 (6.5%) SOT patients had detectable HEV RNA and 8 developed chronic HEV infection. In the Netherlands, Haagsma et al. [273] concluded that SOT recipients had increased risk of developing a chronic HEV infection but that the prevalence of posttransplant infection was low (1-2%). According to Riveiro-Barciela et al. [52], similar to HIV patients, SOT recipients with liver cirrhosis are prone to HEV infection, especially if the cirrhosis appeared after the transplantation. In some cases when the disease evolves to cirrhosis, a retransplantation of the liver is needed [274]. On the contrary, Buffaz et al. [275] concluded that liver transplant patients may not be particularly prone to developing severe HEV infections.

Patients with preexisting liver disease, mainly by other hepatotropic viruses, can also display higher prevalence than the general population [53]. In Albania, seroprevalence among patients with chronic liver disease (HBV, HCV, and HDV infections) was 36.6% versus 12.1% among controls [58]. However, Pischke et al. [54] concluded that HCV/HBC patients did not present higher risk of HEV infections compared to controls. On the other hand, increased severity of infection in this group has been suggested [87]. Blasco-Perrin et al. [276], however, concluded that mortality among HEV patients with and without decompensated chronic liver disease was similar.

Although some authors point out that hemodialysis patients may be a group of risk for HEV [277], the results in Europe are unclear. While some authors reported higher seroprevalence among this group [56, 278], others did not find significant differences when compared to the controls [55, 279, 280].

Patients treated with immunosuppressants are also supposed to be at risk of HEV infection. Pischke et al. [54] reported that patients with autoimmune hepatitis were more likely to test HEV-positive than controls. However, Senosiain et al. [281] found a lower prevalence among patients with inflammatory bowel disease than for the general population. Similarly, Bauer et al. [282] studied the HEV infection in patients with inflammatory arthritides under immunosuppressive therapy, but no chronic infection was observed.

HEV transmission through blood products has been described in France and the UK [283–285]. In fact the screening of blood donors samples in Europe has reported prevalences of viral RNA of 0.03% in Spain [36], 0.045% in France [286], 0.012% in Austria [287], 0.04% in UK [288], 0.076% in the Netherlands [289], or 0.081% in Germany [290]. Although very few cases of transmission by this route have been documented, the active viremia found in donors suggests the possibility of parenteral transmission. Indeed, some authors suggest the need for a systematic screening of blood donations by detecting HEV RNA [291].

In conclusion, according to the published data, HEV is widespread among industrialized countries. Some animal species may act as reservoirs for the infection, preferentially swine, but also wild boar or deer. The main routes of transmission are contact with infected animals and the consumption of contaminated food, usually pork products. Therefore, higher control measures to detect contaminated food and to avoid professional exposure to HEV should be implemented. Blood components can also be a source of virus and should be screened. Although frequently self-limited, infections sometimes evolve to chronic infection in immunosuppressed individuals and this may lead to cirrhosis. There is a need to improve the HEV diagnostic in previously considered virus-free areas, especially among immunocompromised patients with elevated liver enzymes levels and those with preexisting hepatic alterations, as they may suffer faster deterioration of the liver function. Information and advice to prevent infection should also be given to these groups.

## Figures and Tables

**Table 1:** Seroprevalence values reported among general population and blood donors in different European countries.

Country	Group	Number of samples (year)	Assay	Seroprevalence	Reference
UK	GP	1591 (2004)	FD	13%	[25]
		1140 (1991)		13.5%	
Southwest England	BD	487	W	15.8%	[26]
Scotland	BD	1559	W	4.7%	[27]
Ireland	BD	1076	W	5.3%	[28]
Norway	BD	1200	W	14%	[29]
France	BD	10569	W	22.4%	[30]
Southwestern France	BD	512	W	52.2%	[31]
Denmark	BD	504	W	19.8%	[32]
			NIH	10.7%	
Netherlands	BD	5239	W	26.7%	[33]
Spain	GP	1280	BK	7.3%	[34]
Spain	GP	2305	HA	2.17%	[35]
Spain	BD	1082	W	19.97%	[36]
			MK	10.72%	
Germany	GP	1092 (2011)	Ax	34.3%	[37]
		1092 (1996)		50.7%	
Germany	GP	4422	*r*L	16.8%	[38]
Southwest Switzerland	BD	550	MP	4.9%	[39]
			DP	4.2%	
			FD	21.8%	
Greece	BD	265	Adaltis	9.43%	[40]

GP = general population.

BD = blood donors.

Assays: FD = Fortress Diagnostics (identical to Wantai), W = Wantai, NIH = NIH in-house assay, BK = Biokit, HA = HEV Ab, MK = Mikrogen ELISA test, Ax = Axcom, MP = MP Diagnostics, DP/A = Dia.Pro/Adaltis, and *r*L = *recom*Line Mikrogen (Western Blot).

**Table 2:** Risk groups reported in Europe with increased HEV seroprevalence when compared to a control group. Only studies that analyze a control group are included in the table.

Risk factor	Risk group	Country	Seroprevalence		Reference
			Risk group	Control group	
Contact with animals	Pig farm workers	France	44%	26%	[41]
	Forestry workers		36%		
	Forestry workers	France	31.2%	19.2%	[42]
	Swine veterinarians	Netherlands	11%	2%	[43]
	Nonswine veterinarians		6%		
	Farmers	Denmark	50.3%	32.9%	[44]
	Swine farmers	Sweden	13%	9%	[45]
	Swine farmers	Moldova	51.1%	24.7%	[46]
	Animal breeders	Italy	25.6%	0%	[47]
	Slaughterers	Germany	41.7%	15.5%	[48]
	Forestry workers	Germany	18%	11%	[49]

Contact with sewage	Agricultural workers	Turkey	34.8%	4.4%	[50]

Immunosuppression	HIV patients	Italy	6.7%	2.7%	[51]
	HIV patients	Spain	9.2%	3.5%	[52]
	HIV patients with cirrhosis		22.7%		
	HIV patients	Spain	23.5%	12%	[53]
	HIV + HCV patients		40%		
	Autoimmune hepatitis patients	Germany	7.7%	2.0%	[54]
	Hemodialysis patients	Italy	6%^*∗*^	2.7%	[55]
	Hemodialysis patients	UK	36.8%	18.8%	[56]
	Post-liver transplant cirrhosis	Spain	32.1%	3.5%	[52]
	Heart transplant	Germany	11.3%	2%	[57]

Preexisting liver diseases	HCV patients	Spain	23.5%	12%	[53]
	Chronic liver disease patients	Albania	36.6%	12.1%	[58]

^*∗*^No significant difference found.
